# Molecular Basis of Pathogenic Variants in the Fibrillar Collagens

**DOI:** 10.3390/genes13071199

**Published:** 2022-07-04

**Authors:** Allan J. Richards, Martin P. Snead

**Affiliations:** Vitreoretinal Research Group, John van Geest Centre for Brain Repair, University of Cambridge, Forvie Site, Robinson Way, Cambridge CB2 0PY, UK; mps34@cam.ac.uk

**Keywords:** fibrillar collagen, critical amino acids, pathogenic variants

## Abstract

The fibrillar collagen family is comprised of the quantitatively major types I, II and III collagens and the quantitatively minor types V and XI. These form heterotypic collagen fibrils (composed of more than a single collagen type) where the minor collagens have a regulatory role in controlling fibril formation and diameter. The structural pre-requisites for normal collagen biosynthesis and fibrillogenesis result in many places where this process can be disrupted, and consequently a wide variety of phenotypes result when pathogenic changes occur in these fibrillar collagen genes. Another contributing factor is alternative splicing, both naturally occurring and as the result of pathogenic DNA alterations. This article will discuss how these factors should be taken into account when assessing DNA sequencing results from a patient.

## 1. Introduction

The fibrillar collagens consist of the quantitatively major types I, II and III along with the quantitatively minor types V and XI. The major types are differentially expressed with type I being present in bone, skin and tendons; type II collagen is the major collagen present in cartilage and vitreous humour; while type III is expressed in blood vessels and skin. Type V and XI collagen are not as highly expressed as the other three types, but have a regulatory role in controlling fibrillogenesis of the major collagens ([1,2,3,4,5,6] and references therein). Type V is mainly co-expressed with types I and III collagen, while type XI is usually co-expressed with type II collagen. This differential expression and regulatory functions results in distinct clinical outcomes, when pathogenic variants (disease causing changes) occur in the different collagen types, such as osteogenesis imperfecta (type I), various forms of Ehlers–Danlos syndrome (types I, III and V), and chondrodysplasias (types II and XI). Collagen types XXIV and XXVII also form fibrils but have a slightly different structure from the classical fibrillar collagens [4] and will not be discussed here. There are many critical stages and structural constraints in the synthesis of collagen fibrils, which involve large polypeptide sequences, providing many amino acids in these collagen molecules which may be affected by genetic variation, and result in inherited disorders. Most of these pathogenic variants are unique and private to individual families, and, when identified for the first time, need to be assessed to determine their pathogenicity. Understanding the biosynthetic pathway and critical structures of collagen molecules greatly aids this process.

## 2. Biosynthesis of the Fibrillar Collagens

Fibrillar collagens are first synthesised as pro-α collagen chains which consist of a signal peptide sequence necessary for secretion, an amino terminal-propeptide (N-propeptide), a collagen domain and a carboxy terminal propeptide (C-propeptide). The central collagen domain contains a repeating glycine-Xaa-Yaa amino acid sequence (Gly-X-Y) of approximately 338 triplets, where the X position is often a proline and the Y position is often hydroxy proline. The pro-α chains assemble into a trimeric procollagen molecule via the C-propeptide domain which contains a region called the chain recognition sequence, and allows three α chains to assemble in a type specific manner [7]. This initiates the collagen domain to start forming an extended triple helix, which proceeds to zip up in a C to N terminal direction. Prior to helix formation the α chains are subject to post-translational modification, namely, hydroxylation of certain proline and lysine residues [8]. As the triple helix forms, this modification can no longer take place. However, it can still occur in regions of the α chains where the triple helix formation is still incomplete. When the procollagen molecule has formed, it is secreted, during which the N and C propeptide regions are removed from types I, II and III collagen enzymatically at specific sites, leaving only short N and C telopeptides either end of the collagen helical region. In collagen types V and XI the N-propeptides are only partially processed with large regions retained on the collagen molecule [9,10]. The collagen monomer can be either a homo trimer composed of three identical α chains (types II and III) or heterotrimers (types I, V and XI) composed of non-identical α chains synthesised from different genes, the composition of these heterotrimers can also vary between different tissues, and in some tissues trimers containing mixed type V and XI α chains have been observed suggesting that these could be considered as a single class [6] (Table 1).

**Figure 1:**
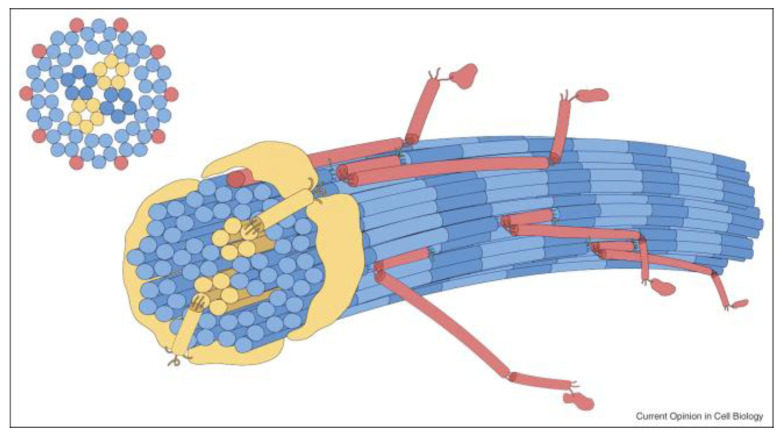
Schematic of the 10 + 4 microfibril structure of a thin cartilage collagen fibril. A pair of collagen XI microfibrils comprise half of a 4 microfibril core surrounded by 10 microfibrils at the surface. The collagen XI/IX/II assembly is a crosslinked heteropolymer, as is V/I, and is an important component of the fibril assembly mechanism. Blue: collagen II molecules; yellow: collagen XI molecules; red: collagen IX molecules. The N-terminal thrombospondin-like domains of collagen XI (yellow) are shown extending from the core microfibrils onto the fibril surface. By K.E. Kadler, Hill, & Canty-Laird, 2008, Current Opinion in Cell Biology, 20: 495–501 [15].

Once the collagen molecules are processed, they self-assemble into collagen fibrils in a quarter staggered manner [13] and are cross linked via certain hydroxy lysine residues [14]. How this self-assembly occurs is still not fully understood, but it is accepted that types V/XI initiate fibril formation and form the core of a collagen fibril to which the more abundant collagen types coalesce Figure 1 [15,16]. The large N-propeptides that are retained on types V/XI regulate fibril diameter, such that in tissues where expression of type V/XI is higher, fibril diameter is thinner. This is particularly important where transparency is important such as the cornea and vitreous. Other molecules such as the FACIT collagens (Fibril Associated Collagens with Interrupted Triple helices; types IX, XII, XIV, XVI, XIX, XX, XXI, and XXII), and small leucine-rich proteoglycans also bind to collagen fibrils and play a role on connecting collagen to other components of the extracellular matrix and regulating spacing between collagen fibrils [17]. Along with variable expression of other extracellular matrix molecules a unique cell type specific connective tissue can be synthesised, where the fibrillar collagens act to resist stretching forces.

## 3. Pathogenic Changes

**Figure 2:**
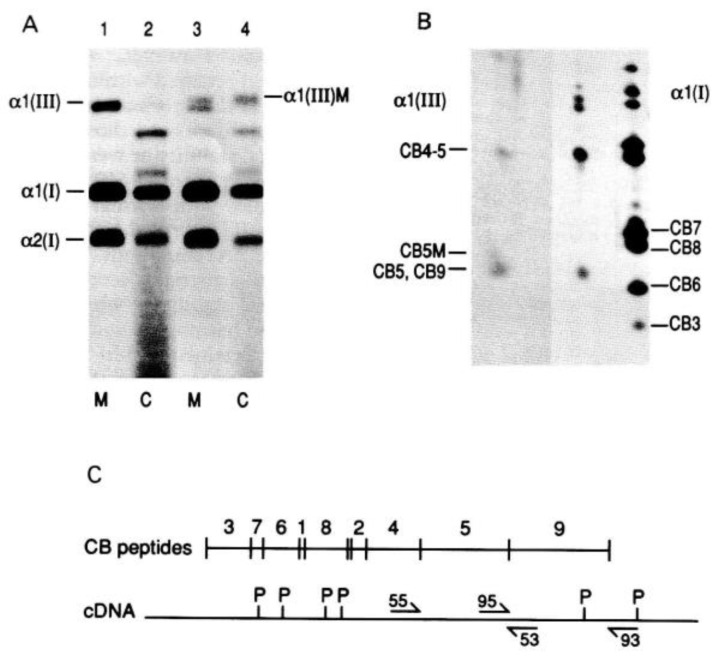
Analysis of type III Collagen from a patient with Vascular Ehlers–Danlos syndrome. Protein analysis. SDS-PAGE analysis of (**A**) radiolabelled collagens, secreted into the medium (M) or retained within the cell layer (C) isolated from a normal control (lanes 1 and 2) and the proband (lanes 3 and 4). Normal (α1(III)) and slow (αl(III)M) migrating type III collagens are shown, as are the two type I collagen α chains (αl(I) and α2(I)). (**B**) Type III collagen cyanogen bromide peptides (αl(III) CB5, CB9, CB4–5) from the proband (lane 1) and a normal control (lane 2). The slow migrating CB5 component is indicated as CB5M. Cyanogen bromide peptides of the αl(I) collagen (lane 3) was used as a standard. (**C**) The arrangement of CB peptides of type III collagen. The position of the oligonucleotides CB93, CB95, CB53 and CB55 are indicated. PstI restriction sites are indicated by P. Reproduced from [J. Med Genet., Richards AJ. et al., 30: 690–693. 1993] with permission from BMJ Publishing Group Ltd. (London, UK).

The earliest studies of abnormal collagens in inherited disorders focused on collagen types I and III. This is because these molecules are expressed in dermal fibroblasts which can be easily grown in vitro, and the collagens studied biochemically, by labelling the cells with ^14^C proline and examining the synthesised collagens by sodium dodecyl sulphate-polyacrylamide gel electrophoresis (SDS-PAGE). Studies using cells from patients with osteogenesis imperfecta (OI) and Ehlers–Danlos Syndrome IV (vascular EDS) demonstrated that types I and III collagen respectively were poorly secreted compared to collagens in cells from unaffected controls [18,19,20]. Furthermore, the secreted collagen from the affected individuals had two forms: one which migrated normally in SDS-PAGE and another that migrated more slowly and was over-modified (Figure 2). 

By digesting type I collagen into fragments with cyanogen bromide it was demonstrated that the over-modification of type I collagen occurred N-terminal to amino acid changes that disrupted the collagen helix in patients with OI [21]. Hence, these changes appeared to delay helix formation at the site of the change, allowing enzymatic modification of the α chains, N-terminal to the altered amino acid sequence, to continue for longer than normal. Historically, this analysis of cyanogen bromide fragments of types I and III collagens was used to target regions of the cDNAs where a pathogenic variant was most likely to occur [22,23,24], as DNA sequencing was not as advanced and as rapid as modern sequencing. However, with the advances in DNA sequencing that have taken place, expertise in this type of collagen protein analysis is scarce and is too time consuming for the high throughput analysis and reporting times now expected from a genetic service. Although, where in silico and family analysis are unhelpful, functional study of these proteins may still help to determine a novel variant’s pathogenicity.

### 3.1. Glycine Substitutions

Because glycine is the only amino acid small enough to fit in the centre of the collagen triple helix, substitution of one of these glycine residues is the most common pathogenic missense change found in the fibrillar collagens, with numerous examples found in nearly all of the genes. These usually have a dominant negative effect, as even a single α chain with a glycine substitution in the collagen trimer will cause helix formation to be delayed, leading to over modification and poor secretion, thus having a negative effect on the α chains synthesised from the “normal” unaltered gene. The resulting phenotype can vary enormously depending on various factors, including the size and nature of the substituting amino acid, the position in the collagen molecule where the substitution occurs and any additional structural abnormality introduced into the collagen molecule that may affect normal processing, fibrillogenesis or interaction with other matrix components or cell receptors [1,25]. For example, some changes have been shown to cause a kink in the collagen molecule, altering the shape of the collagen, so that not only is there reduced collagen secretion, but any abnormally shaped molecule that is secreted also has an additional negative effect on the structure of collagen fibrils [1]. Structural abnormalities to the Gly-X-Y repeat may also result in misalignment of the N-proteinase cleavage site [26,27], resulting in inefficient removal or failure to remove the N-propeptide (see Section 3.7). It has also been found that not only is there reduced secretion and structurally abnormal collagen, but the intracellularly retained molecules can have an adverse effect on cell viability (reviewed in [28]).

The stoichiometry of the different fibrillar collagen molecules can also affect the resulting phenotype. For instance, in types II and III collagens, which are homotrimers i.e., α1(II)_3_ and α1(III)_3_, assuming α chains from the two alleles assemble randomly into trimers only 12.5% will contain three normal α chains. The remaining 87.5% will contain either one, two or three α chains, with the pathogenic change in various positions in the collagen trimer. For heterotrimers such as the α1(I)_2_α2(I) type I collagen the proportion of normal collagen depends upon which α chain contains the pathogenic variant. In type I collagen, pathogenic variants in COL1A1 will affect 75% of molecules, whereas those in COL1A2 will only affect 50% of molecules. Thus, pathogenic variants in COL1A2 tend to result in milder phenotypes than similar ones in COL1A1.

There is also some evidence that over-modified collagen that is secreted can have a different thermal stability compared to the over-modified collagen retained intracellularly [24]. This may reflect a difference in the number of α chains containing a pathogenic variant; however, this is unlikely to be the case for all glycine substitutions in the collagen triple helices.

Determining the pathogenicity of glycine substitutions in types V and XI collagens is not as straightforward. Because they are quantitatively minor compared to type I, II and III, the resulting phenotypes are clinically milder, yet the effects of pathogenic variants can be just as variable. Far fewer of these type V/XI glycine substitutions have been characterised, some of which result in clearly dominantly inherited clinical disorders such as classical Ehlers–Danlos syndrome, type 2 Stickler syndrome and otospondylomegaepiphyseal dysplasia (OSMED) [29,30,31,32]. Other glycine substitutions in *COL11A1* have been found in a more severe disorder namely fibrochondrogenesis [33], but in combination with a second pathogenic variant i.e., with an apparent recessive mode of inheritance. In these cases, the carrier parents did not display the classical phenotype of type 2 Stickler syndrome, and although they had mild myopia, were otherwise considered clinically normal. In our clinic we have seen a similar phenotype in an individual with a glycine substitution in *COL11A1*. This person presented with mild hearing loss and myopia, but without the typical vitreous appearance of type 2 Stickler syndrome, and was not considered to have that disorder (unpublished observation). A similar observation has been made in *COL11A2* where recessive forms of OSMED have been described [34].

Although the α2(V) collagen chain has been detected in the vitreous [35] the exact composition of the type V/XI collagen molecule in the vitreous is uncertain (Table 1), and is complicated by the observation that what was thought to be a novel α3(XI) chain was later found to be a product of the *COL2A1* gene. Examination of the vitreous of a patient with a *COL5A2* glycine substitution and classical EDS II found no vitreous abnormality [29] so it is uncertain how much of a contribution α2(V) collagen makes to type XI collagen in the vitreous.

The current U.K. best practice guidelines for variant classification in rare disease [36] state that glycine substitutions in collagen genes such as *COL1A1* can be upgraded from PM1 (pathogenic moderate 1, located in a mutational hot spot and/or critical and well-established functional domain (e.g., active site of an enzyme) without benign variation) to a strong indication of pathogenicity. It may be more appropriate for collagen types I, II and III that glycine substitutions should be considered as a very strong indication of pathogenicity (PM1 as very strong). However, the effect and mode of inheritance of glycine substitutions in types V and XI are less certain and these may still be classified as only a strong indication of pathogenicity. This classification should not apply to glycine substitutions that happen to occur in the X and Y positions of the collagen triple helix, nor in the amino and carboxy propeptides.

### 3.2. X and Y Position Substitutions

The other common missense pathogenic variants found in the fibrillar collagens are those that change an arginine to cysteine. Cysteine should not occur within the triple helical region of the fibrillar collagens, as they lead to inappropriate disulphide bonding, which can also be detected by SDS-PAGE [20]. As is the case for glycine, these arginine to cysteine substitutions can lead to a wide variety of phenotypes. In type II collagen, these range from premature osteoarthritis to Czech dysplasia [37,38]. There are other examples of X and Y position substitutions [39,40], but none are common and using the current guidelines similar novel variants are unlikely to initially be classified as pathogenic or likely pathogenic without additional family analysis or functional studies. It should not be forgotten that some missense changes have their effect by altering processing of the pre-mRNA (splicing), such as the *COL2A1* c.905C>T p.(Ala302Thr) pathogenic variant which creates a de novo donor splice site, within an exon, that is used preferentially to the natural one [41], so in silico analysis for possible splicing effects should be performed on all variants.

### 3.3. Null Alleles

Any pathogenic variant that alters the reading frame via deletions, insertions or a premature termination codon usually results in the abnormal mRNA transcript being degraded via the nonsense mediated decay pathway [42]. This leads to only 50% of the normal protein being synthesised, without any abnormal protein being produced. Thus, there is no dominant negative effect with these changes, instead they result in haploinsufficiency. The clinical effect is therefore milder than many of the missense changes, resulting in mild forms of OI in type I collagen and typically Stickler syndrome in type II collagen rather than a severe chondrodysplasia such as spondyloepiphyseal dysplasia congenita [43,44]. The exceptions to this are those changes that occur in or close to the last exon, where a termination codon is to be expected. Here these changes result in a shortened or altered carboxy terminal domain [45]. The presence of a null allele would warrant the use of PVS1 using the best practice guidelines for variant classification [36]. However, the mode of inheritance varies for the different collagen genes. While null alleles in *COL1A1*, *COL1A2*, *COL2A1 COL3A1* and *COL5A1* have been shown to result in dominantly inherited disorders [43,46,47], those in *COL11A1* and *COL11A2* result in recessive disorders [33,48]. As yet, haploinsufficiency of *COL5A2* has not been described in an inherited disorder and the phenotype associated with such a change is unknown.

### 3.4. Abnormal Splicing

**Figure 3:**
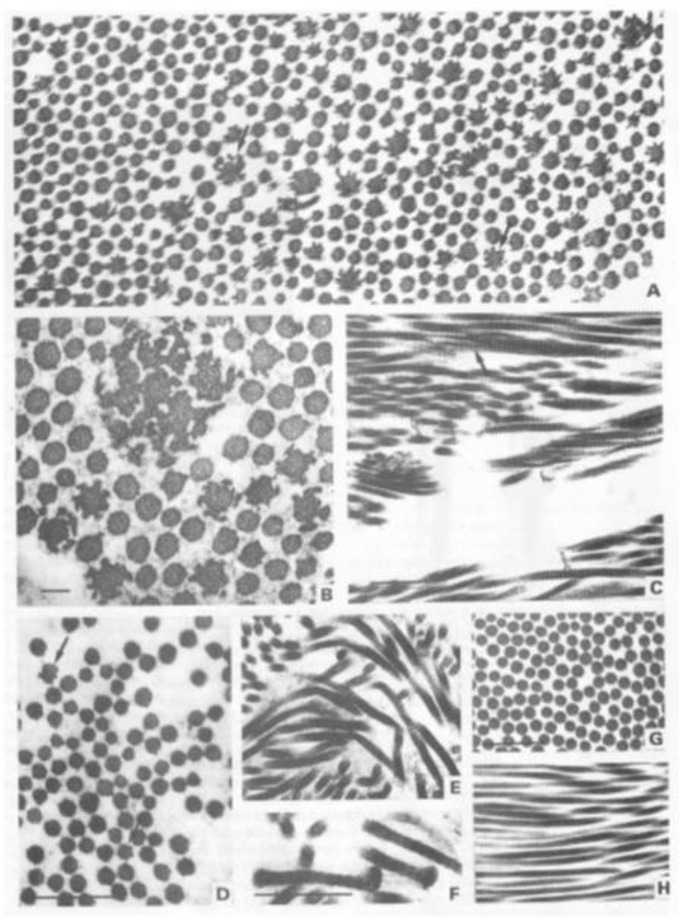
Abnormal collagen fibrils due to exon skipping in *COL5A1*. (**A**–**C**) Election micrographs of skin from the patient’s toe taken at surgery. (**A**) Low power field in transverse section (TS) showing large numbers of collagen fibres with highly irregular profiles. Some fibres form larger arrays with “satellites” extending from the main fibre body producing a cog wheel effect (arrows). Scale bar = 0.2 μm. (**B**) Higher power TS of irregular fibres, some perhaps displaying fusion of smaller fibres to form a massively disordered fibre. Scale bar = 0.1 μm. (**C**) Collagen fibres in longitudinal section (LS) showing a disorganised layering and splaying of fibrils (bold arrows) and a tendency of fibres to basket weave (open arrows). (**D**–**F**) Electron micrographs through dermis from patient’s inner forearm. (**D**) TS of collagen fibres showing some irregular profiles (arrows) but to a much lesser degree than the skin from the toe. Scale bar = 0.5 μm. (**E**) LS of collagen fibres showing marked angularity with sudden changes in direction. Scale bar = 0.5 μm. (**F**) LS of collagen fibres showing stub ends as the fibres abruptly change direction. Scale bar = 0.5 μm. (**G**,**H**) Electron micrographs showing the normal fibre structure and organisation in the dermis. (**G**) In TS, the fibres are smooth and round and fairly regular in size. Scale bar = 0.5 μm. (**H**) In LS, the fibres are closely packed with no angulation and displaying only a limited amount of weaving. Scale bar = 0.5 μm. Reproduced from [J. Med. Genet, Nicholls, A. et al., 33:940–946, 1996] with permission from BMJ Publishing Group Ltd.

The fibrillar collagen genes contain a large number of exons (over 50). Most of these exons encode complete codons for the amino acid sequence. Additionally, those encoding the triple helical domain also encode a complete set of Gly-X-Y triplets, most commonly 54 bp in length or 18 amino acids. This means that a deletion, or skipping of any of these exons during pre-mRNA processing, not only leaves the message in-frame and shortens the resulting α chain, but maintains the repeating Gly-X-Y sequence in register. These shortened α chains will assemble with normal α chains and as for the glycine substitutions have a dominant negative effect. In addition, abnormal molecules containing three shortened chains may be secreted efficiently, as they have a perfect but smaller triple helix. These can then have a dominant negative effect on fibrillogenesis, resulting in abnormal collagen fibrils (Figure 3).

**Figure 4:**
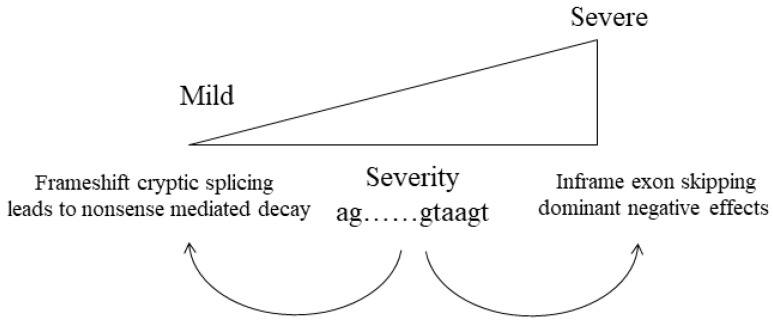
Modification of Clinical Phenotype by Splicing. Disruption to the ag acceptor or gtaagt donor splice sites can result in exon skipping. In collagen genes this typically leaves the message in-frame and the Gly-X-Y amino acid sequence in register, leading to a severe phenotype. In contrast, the use of a cryptic splice site often leads to a shift in the reading frame, nonsense mediated decay and a milder phenotype.

Disruption to the normal splicing signals at the donor, or acceptor splice sites, can result in either exon skipping or a missplicing event that leads to a shift in the reading frame, nonsense mediated decay and a null allele. Theoretically, both could occur from the same pathogenic change, with a variety of differentially splice mRNAs produced from the same allele. This variation could differ between diverse tissues and between individuals with the same pathogenic variant, which may help explain why, for some families, there is a wide range of clinical variability for some of these collagen disorders (Figure 4). 

Similar to the *COL2A1* c.905C>T p.(Ala302Val) example (see above) which results in missplicing [41], silent changes (those that alter a codon but not the amino acid) have also been found to alter normal splicing of the pre-mRNA [49]. As such, these alterations should not be discounted because they do not apparently alter the protein sequence. When analysis of whole gene sequencing is available, it may also be possible to detect deep intronic pathogenic variants. In most of these cases, the deep intronic change creates a de novo acceptor or donor splice site which is used either in preference or in addition to the naturally occurring one [50].

Certain exons in the *COL2A1 COL11A1* and *COL11A2* genes are alternatively spliced [51,52], that is expressed differently in various tissues. When pathogenic variants occur in these alternatively expressed exons the resulting phenotype will differ from those that are within constitutively expressed exons. This is because those pathogenic variants are removed from the mature transcript in tissues where those exons are not expressed. The most commonly seen are those in exon 2 of *COL2A1* which is expressed in the eye but not in mature cartilage. This explains a predominantly ocular form of Stickler syndrome that was often confused with Wagner syndrome, a vitreo-retinopathy without systemic features [53]. Another example are pathogenic variants in exon 9 of *COL11A1*. Here, alternative splicing removes the variant from mature cartilage and modifies the phenotype from the recessive and usually lethal fibrochondrogenesis, to a recessive form of type 2 Stickler syndrome with profound hearing loss [54].

The current best guidelines only allow for variants in the +/− 1 and 2 positions of the donor and acceptor splice sites to be classified as PVS1 [36]. However, there are numerous examples of variants in the +3 to +5 position of donor splice sites that result in abnormal splicing. Although in silico analysis can indicate those that are likely to affect normal splicing, in most of these cases, it is necessary for additional family analysis or functional splicing assays to help determine pathogenicity of these changes.

### 3.5. In-Frame Insertions and Deletions

There are many examples of small and large in-frame deletions and insertions occurring within the triple helical region of the fibrillar collagen genes [43,55]. Like the glycine substitutions and exon skipping variants, these changes will have a dominant negative effect by co-assembling with α chains expressed from the normal allele. The best practice guidelines allow for the use of PM4 criteria [36] when the insertion or deletion occurs within a non-repeat region. However, the collagen triple helix is a repeat region of over 300 Gly-X-Y repeats. Because the length of the collagen helical region is critical for normal collagen biosynthesis and secretion, it is acceptable to use the PM4 criteria for in-frame deletions and insertions within the collagen triple helix. It may even be appropriate to classify these as a very strong indication of pathogenicity.

### 3.6. C-Propeptide Changes

The C-propeptide region of collagens are critical for normal collagen biosynthesis and there are many examples of pathogenic variants occurring in these regions. There is a high degree of homology between the different C-propeptides of the fibrillar collagens. The most critical of the highly conserved residues are eight cysteines which are essential for correct folding of the region prior to α chain assembly into trimers [56]. Alteration of any of these cysteines or creation of additional cysteines within the region disrupts normal folding and therefore this type of change can be considered as a strong indication of pathogenicity using the PM1 criteria. Using PM1 as a strong indication of pathogenicity can also be applied to variants that alter the two conserved amino acids (Alanine-Aspartic acid) in the recognition sequence for the C-proteinase enzyme. In type I collagen, alteration of either of these two amino acids results in a high bone mass form of osteogenesis imperfecta [57] as the altered collagen will be secreted but the C-propeptide will not be removed.

For novel missense changes in the C-propeptide, it may be possible to assign PM1 (as moderate) by comparison to similar, previously characterised pathogenic variants, in regions of high homology that exist between the different collagen types and have been mapped onto the type III collagen C-propeptide crystal structure [58,59].

### 3.7. N-Propeptide and Signal Peptide Sequence Changes

Unlike the C-propeptides, the N-propeptides can vary markedly between the different α chains, containing either a von Willebrand factor C domain, a thrombospondin-1 N-terminal domain or, in the case of proα2(I), neither [4]. Consequently, it is more difficult to determine if a new variant in these regions is pathogenic based upon known critical amino acids. One well-established critical region is the site for the N-proteinase cleavage of type I collagen. Removal of exon 6 from either of the *COL1A1* or *COL1A2* genes either by exon skipping or genomic deletion removes the site for procollagen N-proteinase and a critical cross linking lysyl residue [60] resulting in the arthrochalasia form of EDS (EDS VII).

The N-propeptides also contain an additional short collagenous triple helical Gly-X-Y domain. Although some substitutions of glycine have been documented within this domain for both α1(II) and α1(V) their significance is uncertain. In *COL2A1*, the resulting phenotype can vary markedly between individuals ([49] and unpublished observations]), and in *COL5A1* the c.1588G>A p.(Gly530Ser) variant is now thought unlikely to be pathogenic [61]. Therefore, using PM1 as criteria for pathogenicity with these N-propeptide glycine substitutions should remain as moderate evidence.

The signal peptide starts with the initiating methionine (for translation) and a run of 16–30 hydrophobic amino acids, which target the protein for secretion. Variants which alter the initiating methionine have been documented and are strong evidence for pathogenicity (PVS1) [36,49,62]. In addition, variants within the hydrophobic signal peptides of both α1(I) and α1(V) collagen, which substitute leucine or glycine residues with non-hydrophobic residues, have been shown to inhibit secretion of types I and V collagen [63,64]. However, these needed additional in silico and functional studies to demonstrate pathogenicity.

## 4. Conclusions

In summary, the biosynthesis of the fibrillar collagens is highly regulated with many critical structures required for normal assembly, secretion and fibrillogenesis. Most pathogenic variants that occur in these genes are rare or private to a single family, so analysis of a patient’s DNA often results in a novel sequence variant. However, as these collagens are some of the most abundant in the human body, the study of their protein structure and genes occurred relatively early in the characterisation of genetic disorders. Consequently, there are numerous studies that provide evidence for the type of sequence variants which typically result in inherited disorders of the fibrillar collagens and, in combination with the best practice guidelines [36], should aid in the classification of novel variants.

## Figures and Tables

**Table 1:** The Fibrillar Collagens.

Collagen Type	Stoichiometry	Tissue	Genes
Type I	α1(I)_2_α2(I)	Multiple tissues including Bone, Skin, Tendon	*COL1A1*, *COL1A2*
Type II	α1(II)_3_	Cartilage, Vitreous	*COL2A1*
Type III	α1(III)_3_	Multiple tissues including Skin, Blood vessels	*COL3A1*
Type V	Major form α1(V)_2_α2(V) Placenta α1(V)α2(V)α3(V)	Co-expressed with Types I and III collagens	*COL5A1* *COL5A2* *COL5A3*
Type XI	Cartilage α1(XI)α2(XI)α3(XI) Vitreous α1(XI)_2_α3(XI) ?? α1(XI)_2_α2(V) ??	Co-expressed with Type II collagen	*COL11A1* *COL11A2* *COL2A1* *COL5A2*

Stoichiometry and genes encoding the different fibrillar collagens. Note: The α3(XI) chain of type XI collagen was found to be a modified product of the *COL2A1* gene [11,12]. ?? The exact composition of the type V/XI collagen molecules in the vitreous is uncertain, see Section 3.1.
